# Shunt rate of ductus arteriosus and ductus venosus in middle and late fetuses and their application value in the evaluation of fetal growth restriction

**DOI:** 10.12669/pjms.39.6.7649

**Published:** 2023

**Authors:** Yijia Zhou, Qunqun Chen, Ruhui Luan, Yaping Zhao

**Affiliations:** 1 Yijia Zhou, Department of Ultrasound, The Second Affiliated Hospital of Wenzhou Medical University, Wenzhou 325100, Zhejiang Province, P.R. China; 2 Qunqun Chen, Department of Ultrasound, The Second Affiliated Hospital of Wenzhou Medical University, Wenzhou 325100, Zhejiang Province, P.R. China; 3 Ruhui Luan, Department of Ultrasound, The Second Affiliated Hospital of Wenzhou Medical University, Wenzhou 325100, Zhejiang Province, P.R. China; 4 Yaping Zhao, Department of Ultrasound, The Second Affiliated Hospital of Wenzhou Medical University, Wenzhou 325100, Zhejiang Province, P.R. China

**Keywords:** Doppler ultrasound, Ductus venosus, Ductus arteriosus, Fetal hemodynamics, Fetal growth restriction

## Abstract

**Objective:**

To explore the Shunt rate of ductus arteriosus (DA) and ductus venosus (DV) in middle and late fetuses and their application value in the evaluation of fetal growth restriction (FGR).

**Methods:**

In this retrospective observational study, we reviewed the clinical data of the patients who admitted to the Second Affiliated Hospital of Wenzhou Medical University from September 10, 2017 to November 27, 2018, and finally included 44 normal women at 28-31 weeks of pregnancy (Normal group) and 15 pregnant women with fetal growth restriction (FGR) within 28-31 weeks of gestation (FGR group). We measured blood flows of the DA (Q_DA_), pulmonary artery (QP_A_), DV (Q_DV_), and umbilical vein (Q_UV_) and the shunt rates of the DA and DV (Q_DA_/Q_PA_ and Q_DV_/Q_UV_, respectively) in all fetuses. We compared the mean variables between groups using the Normal group means as the normal reference values for analysis.

**Results:**

DA shunt rate was linearly and positively correlated with gestational age (Y=1.455X+2.787; r=0.767, *P*<0.01), while the DV shunt rate was linearly and negatively correlated with gestational age (Y=-2.791X+126.885; r=0.761, *P*<0.01). The DA shunt rates (Q_DA_/Q_PA)_ of fetuses in the normal were higher than those in the FGR groups, but the differences between the two groups were not statistically significant (*P* > 0.05). The DV shunt rates (Q_DV_/Q_UV_) of fetuses in the normal were significantly lower than those in the FGR groups (*P* < 0.05). The DV shunt rates in the FGR group were significantly higher than those in the normal group with differences being statistically significant at 30-30^+6^ and 31-31^+6^ gestational weeks (*P* < 0.05) The receiver operating characteristic curve (ROC curve) showed that the higher the shunt rate, the worse the birth outcome of a fetus with FGR.

**Conclusions:**

The DV shunt rate in middle- and late-stage fetuses can predict the fetal birth outcome, and the higher the shunt ratio, the worse the birth outcome of FGR fetuses.

## INTRODUCTION

The ductus arteriosus (DA) and ductus venosus (DV) are unique vascular structures during the fetal period. Their hemodynamic and morphological changes reflect the fetal hemodynamic state in utero.^1^,^2^ The blood flow and shunt rates of the fetal DA and DV are affected by diverse factors, such as maternal pregnancy complications, fetal developmental abnormalities, placental abnormalities, and others, which may lead to poor fetal prognoses. Therefore, monitoring the fetal DA and DV shunt rates and the Doppler blood flow spectrum before birth is important.^2^,^3^

The normal reference value ranges of fetal DA and DV blood flows have been defined,^4^,^5^ but few reports on DA and DV shunt rates exist. Ultrasound variables have been used to estimate fetal body mass, abdominal circumference and growth rate indicators;^6^ but, its value for diagnosing and predicting fetal growth restriction (FGR) and the perinatal prognosis of high-risk fetuses is unclear. The DV blood flow and shunt rate are mostly used to evaluate fetal distress during pregnancy, severe pre-eclampsia and intrauterine hypoxia.^7^,^8^ However, whether DA and DV shunt rates can be used for assessing intrauterine growth and development in fetuses with FGR and for predicting the postnatal outcome remains unclear. Therefore, we conducted this study to obtain accurate measurements that should be comprehensively analyzed for the clinical evaluation and prediction of FGR.

## METHODS

In this retrospective observational study, we reviewed the clinical data of the patients who were admitted to the Second Affiliated Hospital of Wenzhou Medical University from September 10, 2017 to November 27, 2018. A total of 44 normal women at 28-31 weeks of pregnancy were included as Normal group (age, 19-42 years with a mean of 29.66 ± 5.19 years) and 15 pregnant women with fetal growth restriction (FGR) within 28-31 weeks of gestation were included as FGR group (age 23-40 years with a mean of 30.70 ± 5.62 years). The patients in the Normal group were divided into four groups according to the gestational weeks of the fetus: 28 weeks (Group-1, *n*=12), 29 weeks (Group-2, *n*=10), 30 weeks (Group-3, *n*=11), and 31 weeks (Group-4, *n*=11). FGR occurs when the fetal growth does not reach its genetic potential due to the influence of pathological factors (maternal, fetal, or placental diseases) and it is manifested as ultrasonographic estimated fetal weight or abdominal circumference values lower than the 10^th^ percentile of the corresponding fetal age.^9^

### Inclusion criteria for the FGR group:

- Singleton pregnancy
- Regular menstruation before pregnancy, with documented last menstruation and accurate gestational week estimation
- Fetal weight estimated by ultrasound lower than the 10^th^ percentile of the corresponding fetal age (with the NICHD Asian fetal growth curve as the standard reference).^10^ Fetal head circumference, abdominal circumference and femur length values lower than two standard deviations of the normal mean value
- Clear pathological factors (maternal, placental, or umbilical cord diseases)
- Continuous observation for more than two weeks and birth in our hospital
- Fetal birth weight was lower than 2500 g after 37 weeks of gestation.

### Inclusion criteria for the Normal group:

- Singleton pregnancy
- Regular menstruation before pregnancy, with documented last menstruation and accurate gestational week estimation
- Pregnant women who were in good health and had no pathogenic factors for FGR
- Perinatal ultrasound showed that the fetal development was consistent with the gestational age, without structural abnormalities, and the placenta and amniotic fluid volume were normal
- Fetal birth weight between 2500g and 4000g

### Exclusion criteria:

- Twin or multiple pregnancies
- Irregular menstruation before pregnancy, impossibility to accurately verify gestational age
- Fetal size estimated by ultrasound consistent with the gestational age, and fetal weight higher than the 10^th^ percentile of the corresponding gestational age
- Fetus complicated with serious malformations, such as chromosomal karyotype abnormalities, congenital heart disease, and others
- Absence of definite pathological factors as the cause of FGR
- Impossibility to obtain accurate fetal body position due to obesity of the pregnant woman
- Incomplete follow-up data

One minute APGAR determines how well the baby tolerated the birthing process.^11^ We used one minute APGAR values at birth, to classify the newborns with FGR into a normal group (10 points, seven cases, mean 36.14±1.07 weeks, average birth weight 2225.14±234.40g) or an FGR group (< 10 points, eight cases, average 34.13±0.84 weeks, average birth weight 1846.38±170.24g).

### Ethical Approval

The ethics committee of our hospital approved this study (No. 2022-K-159-02, Date: October 27, 2022).

### Instruments and methods

**Fig.1:**
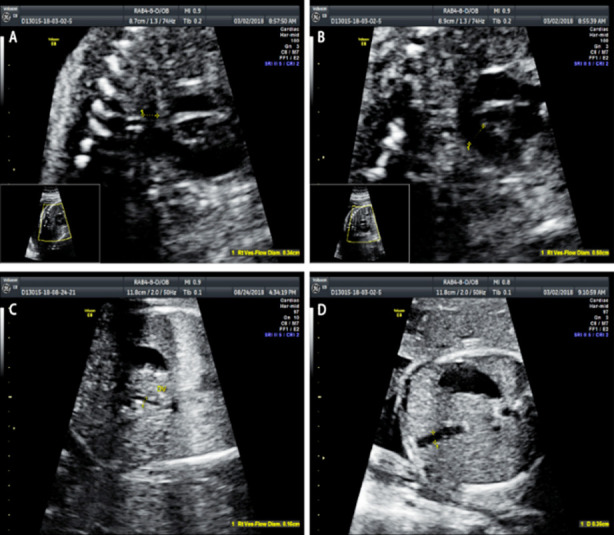
2D image measurement of inner diameter A) D_DA_; B) D_PA_; C) D_DV_; D) D_UV_.

**Fig.2:**
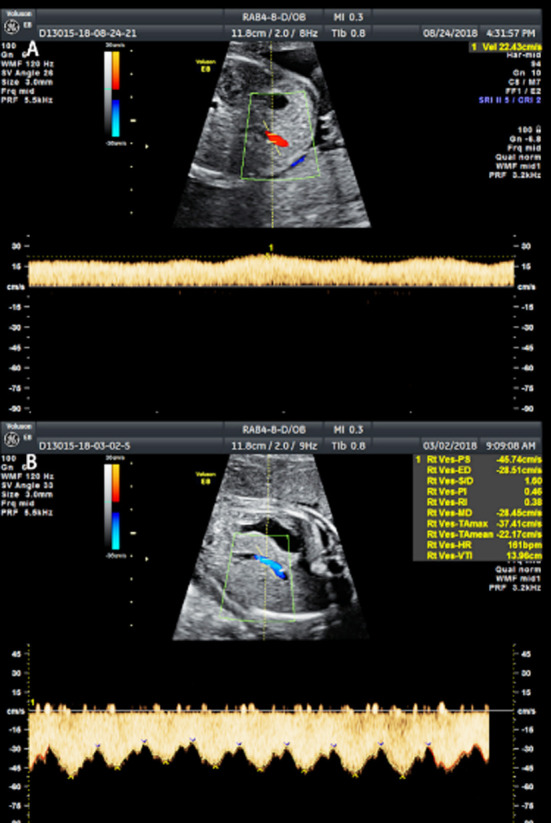
Maximum flow velocity measured by blood flow spectrum A) Vmax-_DV_; B) Vmax-_UV_.

**Fig.3:**
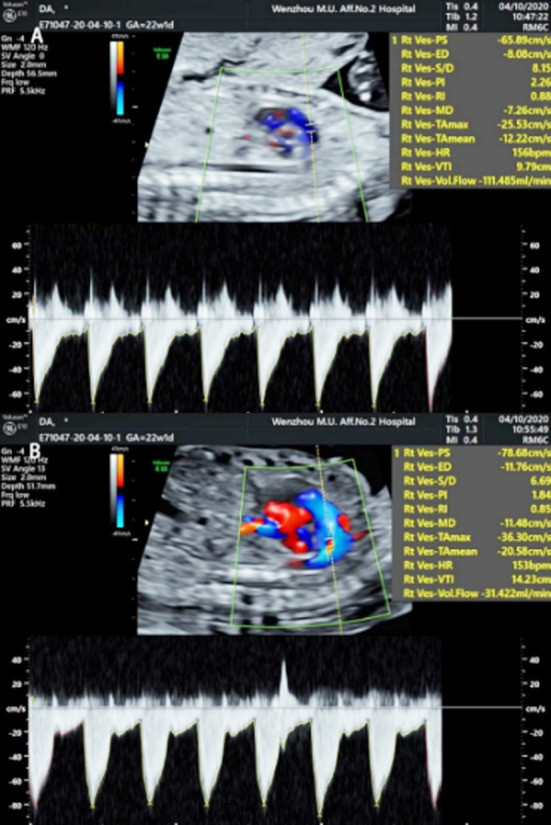
VTIPA of blood flow spectrum measurement A) PA; B) DA.

We used a GE Voluson E8 color Doppler ultrasound diagnostic instrument to take measurements, with a rab4-8-d probe at a frequency of 4-8 MHz. We measured the biparietal diameter, head circumference, abdominal circumference and femur length of the fetuses applying obstetric procedures to check the gestational weeks and observe the growth and development of the fetuses with their pregnant mothers in a supine position. We performed overall examinations to allow us to identify fetal abnormalities, we used two-dimensional ultrasound images to evaluate fetuses with abnormal frequency of movements by color Doppler technology measuring the inner diameters of DA, PA, DV and UV (D_DA_, D_PA_, D_DV_ and D_UV_) (Fig.1) and the peak flow velocities of DV and UV (V_max-DV_, V_max-UV_) (Fig.2), the velocity time integrations of PA and DA (VTI_PA_, VTI_DA_) (Fig.3) and the heart rates (HRs). Finally, we calculated the blood flow of PA, DA, UV and DA (Q_PA_, Q_DA_, Q_UV_, Q_DV_) based on the following equations: Q_PA_= (D_PA_/2) ²×π×VTI_PA_ ×HR, Q_DA_= (D_DA_/2)²×π×VTI_DA_×HR, Q_UV_= (D_UV_/2) ²×π×Vmean-_UV_, Vmean-_UV_=1/2 (Vmax-_UV_), Q_DV_= (D_DV_/2) ²×π×Vmean-_DV_, and Vmean-_DV_=0.7 (Vmax-_DV_) .^12^,^13^ The DA shunt rate was defined as Q_DA_ /Q_PA_, and the DV shunt rate by Q_DV_ /Q_UV_.

### Statistical Analysis

We used the SPSS 20.0 statistical software for data analysis, testing for normality with the Shapiro Wilk tests. We expressed measurement data as means±standard deviations (*χ̅*±*S*), and t-test was used to compare the differences between two independent samples. Pearson’s correlation and linear regression analysis were used to analyze the relationship between DA shunt rates or DV shunt rates and gestational age. We obtained a receiver operating characteristic curve (ROC curve) to predict the fetal birth outcomes, and we calculated the sensitivity, specificity and Youden index. *P* < 0.05 means that a difference is statistically significant.

## RESULTS

**Fig. 4:**
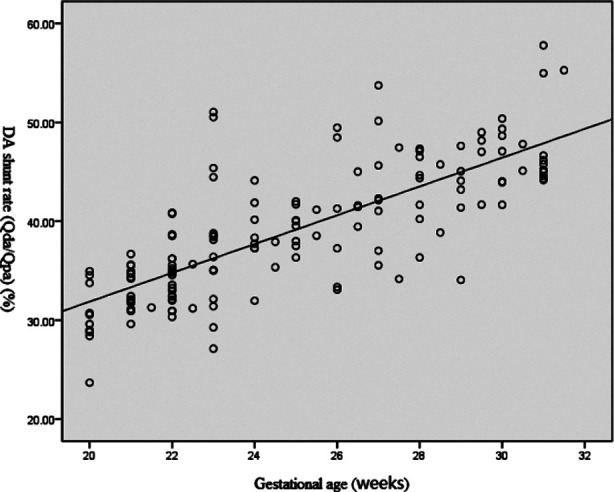
Correlation analysis of DA shunt rate and gestational age.

**Fig. 5:**
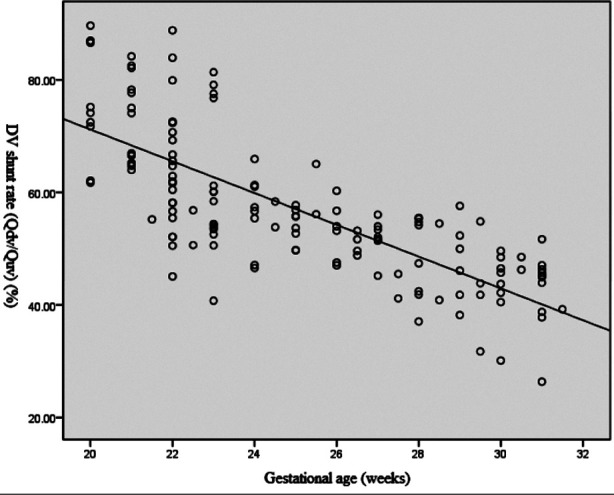
Correlation analysis of DV shunt rate & gestational age.

Correlation analysis results of DA shunt rates, DV shunt rates and gestational age: It was shown that DA shunt rate was linearly and positively correlated with gestational age (Y=1.455X+2.787; r=0.767, *P*<0.01) (Fig. 4), while the DV shunt rate was linearly and negatively correlated with gestational age (Y=-2.791X+126.885; r=0.761, *P*<0.01) (Fig.5).

Comparison of DA shunt rates or DV shunt rates between normal group and FGR group: The DA shunt rates (Q_DA_/Q_PA)_ of fetuses in the normal were higher than those in the FGR groups, but the differences between the two groups were not statistically significant (*P* > 0.05) (Table-I). The DV shunt rates (Q_DV_/Q_UV_) of fetuses in the normal were lower than those in the FGR groups, and the differences between the two groups were statistically significant (*P* < 0.05) (Table-II).

**Table-I:** Comparison of fetal arterial catheter shunt rate (Q_DA_/Q_PA_) between normal group and FGR group (*χ̅*±*S*).

Gestational week	Group	N	Q_DA_/Q_PA_ (%)	t	P
28~28^+6^	Normal group	12	43.27±3.80	0.039	0.845
	FGR group	15	41.04±5.59		
29~29^+6^	Normal group	10	44.12±4.43	0.478	0.400
	FGR group	15	42.92±6.39		
30~30^+6^	Normal group	11	46.19±2.85	0.662	0.427
	FGR group	15	43.27±5.47		
31~31^+6^	Normal group	11	48.16±5.13	0.662	0.427
	FGR group	15	47.12±4.14		

**Table-II:** Comparison of fetal venous catheter shunt rate (Q_DV_/Q_UV_) between normal group and FGR group (*χ̅*±*S*).

Gestational week	Group	n	Q_DA_/Q_PA_ (%)	t	P
28~28^+6^	Normal group	12	48.38±7.24	18.336	<0.001
	FGR group	15	73.10±3.16		
29~29^+6^	Normal group	10	45.83±7.96	6.120	0.024
	FGR group	15	62.87±3.44		
30~30^+6^	Normal group	11	44.17±5.71	5.200	0.035
	FGR group	15	60.05±1.63		
31~31^+6^	Normal group	11	43.17±5.91	6.703	0.018
	FGR group	15	61.09±2.22		

**Fig.6:**
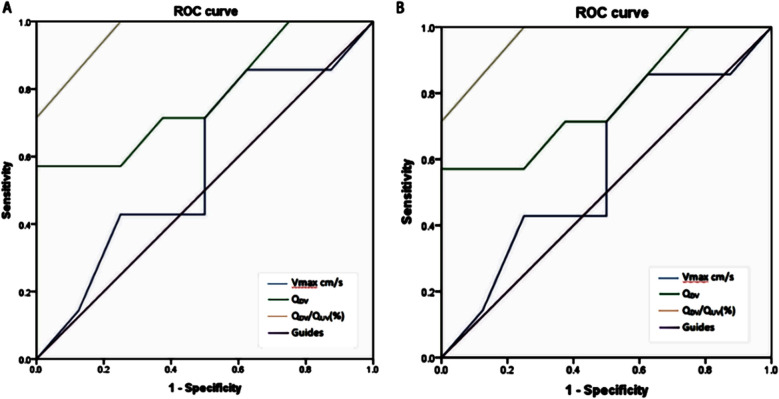
Roc curve of A) fetal ductus venosus blood flow variables predicting abnormal fetal birth outcome at 30 weeks of FGR; B) 31-week FGR fetal venous catheter blood flow parameters predicting abnormal fetal birth outcome.

Analysis between DV shunt rates and FGR: The DV shunt rates of fetuses with FGR (Q_DV-_FGR and Q_UV-_FGR) were higher than those of fetuses in the normal group with differences being statistically significant at 30-30^+6^ and 31-31^+6^ gestational weeks (*P* < 0.05) (Table-III). We generated a receiver operating curve (ROC curve) to depict the association between fetal birth outcome and DV shunt rate at 30-31weeks of gestation in fetuses with FGR. The area under the DV shunt rate (Q_DV-_FGR/Q_UV-_FGR) curve for fetuses with FGR at 30 weeks was 97%; and with DV shunt rate (Q_DV_/Q_UV_) > 60%, the sensitivity, specificity and Youden index for predicting abnormal fetal birth outcomes were 100%, 88%, and 0.88, respectively (Fig.6A). In addition, the area under the curve for the DV shunt rate (Q_DV-_FGR/Q_UV-_FGR) of fetuses with FGR at 31 weeks of gestation was 96%; and, with a DV shunt rate (Q_DV_/Q_UV_) > 60%, the sensitivity, specificity, and Youden index for predicting abnormal fetal birth outcomes were 100%, 75%, and 0.75, respectively (Fig.6B).

**Table-III:** Comparison of DV shunt rate Q_DV-FGR_/Q_UV-FGR_ (%) between normal group and abnormal group with FGR outcome at 28~31^+6^ weeks (*χ̅*±*S*).

Gestational week	DVshunt rate Q_DV-FGR_/Q_UV-FGR_ (%)		t	P

	Normal group (n=7)	FGR group (n=8)		
28~28^+6^	75.89±1.68	70.83±2.15	0.875	0.377
29~29^+6^	65.69±1.88	61.15±2.02	0.026	0.873
30~30^+6^	61.47±1.53	59.12±1.88	9.225	0.016
31~31^+6^	63.24±1.79	59.65±1.89	5.413	0.048

## DISCUSSION

The clinical study of the DV and DA by ultrasound has mainly focused on the correlation between blood flow spectra characteristics and the presence of fetal distress, fetal heart abnormalities, chromosomal abnormalities, FGR, fetal anemia, and others; however, the meaning of diverging shunt rates of the DV and DA remains unclear.

DA blood flow measurements can identify DA stenoses or premature closures and aneurysms that affect the prenatal diagnosis of DA-dependent congenital heart diseases.^14^ International studies have shown that the DA blood flow is positively correlated with the gestational age,^15^,^16^ but there are few studies on its shunt rate. In this study, the DA shunt rate (Q_DA_/Q_PA)_ was positively correlated with gestational age, and the DA shunt rate of fetuses from gestational weeks 28 to 31 was approximately 40%. The demands of fetal growth and development increase gradually during gestation. The DA is responsible for diverting part of the blood flow from the pulmonary artery to the aorta, and supplying the fetal growth together with the blood from the left heart. Therefore, its blood flow also increases gradually during gestation.

Our results showed that DV shunt rate (Q_DV_/Q_UV_) was negatively correlated with gestational age, a finding also reported by Zytoon et al.^5^ and Kiserud et al.^12^ In addition, the DV shunt rate of fetuses with FGR is higher than that of normal fetuses, a result consistent with that of Ritter et al.^17^ Their study showed that when the fetus is ischemic and hypoxic, the proportion of blood flow of the UV entering the DV increases to 90%. Under pathological conditions, such as during abnormal fetal heart development, fetal growth restriction, fetal chromosomal abnormality, intrauterine hypoxia, and other changes that affect the structure and function of the heart; abnormal vascular development or blood composition can cause fetal protective hemodynamic adaptations, which directly affect the shape and blood flow spectra and waveforms of the DV, its blood flow, and the shunt rate. The vascular diameter, portal vena cava pressure difference, and blood viscosity can all affect the DV shunt rate.^18^ The “brain protection effect” causes the blood vessels of important organs like the brain and heart to actively expand in fetuses with FGR; the DV is also actively regulated and gets expanded, so that the blood flow rate of UV entering the DV increases to meet the demands of the left heart.^19^

In this study, we analyzed the value of the DV shunt rate of fetuses with FGR at 28-31 weeks for predicting the fetal birth outcome, and we found that the shunt is statistically significant during some gestational weeks (*P* < 0.05), a study with a large study group should confirm our finding. We cannot rule out the possibility that the DV shunt in fetuses with FGR (Q_DV-FGR_/Q_UV-FGR_) needs to attain a certain threshold to become predictive of birth outcomes. At the same time, the DV shunt in fetuses with FGR (Q_DV-FGR_/Q_UV-FGR_) may only be useful to predict birth outcomes in fetuses older than 30 weeks. Our receiver operating curve (ROC curve) to predict the fetal birth outcomes suggests that DV shunt rate (Q_DV-FGR_/Q_UV-FGR_) > 60% beyond the 30 weeks of gestation is abnormal and should prompt close monitoring. These results are consistent with those of Ritter et al.^17^ which showed that the DV shunt rate (Q_DV_/Q_UV_) of fetuses with FGR is significantly increased and that the higher the rate, the worse the birth outcome. In addition, Wang B et al.^20^ assessed the risk of FGR through the combined screening of early and mid-term pregnancy, the results showed that 33 cases of FGR were screened and 1507 normal pregnant women were selected, in the second trimester of pregnancy, BMI, uterine artery pulsatility index (UTA-PI) and umbilical artery pulsatility index (UA-PI) were risk factors for FGR, of which UTA-PI was the most dangerous. Including more factors to evaluate FGR is also the direction of our team’s research in the future.

### Limitations

Because DV is trumpet shaped and the vessel diameter is thin, the inner diameter of the middle DV of 20-31weeks fetuses measured in this study was between 1.0 and 2.4 mm, making accurate measurements difficult.^21^ Moreover, the trumpet-shaped anatomical characteristics complicate DV hemodynamics. Therefore, the accuracy of the formula for calculating the blood volume needs further study. Secondly we failed to conduct detailed grouping analyses in this study due to the small sample sizes of the different subgroups of gestational age within the FGR group and the diverse factors affecting the fetal birth outcomes of FGR. Larger studies are needed to assess the correlation between the DV shunt rate and fetal birth outcomes.

## CONCLUSION

The DV shunt rate in middle and late-stage fetuses can predict the birth outcome, and the higher the shunt ratio, the worse the birth outcome of FGR fetuses.

### Authors’ contributions:

**YZ:** Conceived and designed the study.

**QC, RL** and **YZ:** Collected the data and performed the analysis.

**YZ**: Was involved in the writing of the manuscript and is responsible for the integrity of the study.

All authors have read and approved the final manuscript.
